# Renal Fat Accumulation Assessed by MRI or CT and Its Association with Clinical and Metabolic Disorders: A Systematic Imaging Review

**DOI:** 10.3390/jcm14124305

**Published:** 2025-06-17

**Authors:** Hadar Raphael, Eyal Klang, Eli Konen, Avshalom Leibowitz, Yael Frenkel-Nir, Sara Apter, Ehud Grossman

**Affiliations:** 1 Department of Imaging, The Chaim Sheba Medical Center, Tel Hashomer 5265601, Israel; aharonith@gmail.com (H.R.); eli.konen@sheba.health.gov.il (E.K.); saraapter@gmail.com (S.A.); 2 Faculty of Medicine, Tel Aviv University, Tel-Aviv 6997801, Israel; avshalom.leibowitz@sheba.health.gov.il (A.L.); yael.frenkelnir@sheba.health.gov.il (Y.F.-N.); 3 The Windreich Department of Artificial Intelligence and Human Health, Icahn School of Medicine at Mount Sinai, Mount Sinai Health System, New York, NY 10029, USA; eyalkla@hotmail.com; 4 Internal Medicine D, The Chaim Sheba Medical Center, Tel Hashomer 5265601, Israel; 5 Medical Management Department, The Chaim Sheba Medical Center, Tel Hashomer 5265601, Israel; 6 Adelson Medical School, Ariel University, Ariel 4077625, Israel

**Keywords:** fatty kidney, MRI, CT, inflammation, obesity, fatty liver, diabetes mellitus, hypertension

## Abstract

**Background:** The term “fatty kidney” refers to the accumulation of fat within the kidney. There is no clear definition of a fatty kidney. In our previous study, we defined a fatty kidney as one with fat accumulation of more than 4% in the kidney as detected by magnetic resonance imaging (MRI). This condition is associated with renal inflammation and contributes to the development of kidney dysfunction. Fat accumulation in the kidney can be detected using imaging modalities such as computed tomography (CT) or MRI. Given the clinical importance of renal fat deposition, the aim of this review was to investigate how imaging findings in this condition correlate to disease prevalence and metabolic disorders. **Methods**: A systematic review was conducted in accordance with the PRISMA guidelines. The databases searched included PubMed, Scopus, Web of Science, and Cochrane Library up to August 2024. Studies employing MRI or CT for renal fat quantification were included. Data were extracted, and their quality was assessed using the QUADAS-2 tool. **Results**: Twenty-eight studies comprising 6994 participants met the inclusion criteria. Most studies used MRI (75%) for fat quantification, with CT limited to renal sinus evaluation. Renal fat fractions (FFs) ranged from 0.4% to 55.3%, with higher values consistently observed in individuals with obesity, diabetes, chronic kidney disease, and hypertension. A consistent positive association was observed between fatty kidney and fatty liver, suggesting shared pathogenic mechanisms. **Conclusions**: Fatty kidney appears to be a distinct and clinically relevant entity with strong links to metabolic dysfunction. Imaging-based quantification—particularly MRI—offers a promising tool for early detection, yet standardization is needed. The findings underscore the need for further research into fatty kidney as a modifiable risk factor for renal and cardiovascular disease.

## 1. Introduction

The term “fatty kidney” refers to the excessive accumulation of lipids within renal tissues, including the renal sinus and parenchyma. This emerging condition has attracted attention because of its potential role in the progression of chronic kidney disease (CKD), hypertension, and other metabolic disorders. Fatty kidney is believed to impair renal function by exerting physical pressure on vascular and structural components of the kidney, leading to altered hemodynamic and metabolic stress and inflammation. Chronic low-grade inflammation is believed to contribute significantly to the development and progression of CKD [1]. Increasingly recognized for its clinical significance, fatty kidney is now being studied as an important entity with potential implications for diagnosis and treatment [2,3,4].

Imaging modalities such as magnetic resonance imaging (MRI) and computed tomography (CT) play a central role in the diagnosis of fatty kidney. CT provides excellent spatial resolution for assessing lipid content in the renal sinus. MRI, particularly with advanced techniques such as dual-echo sequences and the Dixon technique, enables detailed quantification of renal fat fractions, offering a non-invasive method to explore lipid deposition in the renal parenchyma. These modalities, however, have been applied inconsistently across different studies, with no standardized methodologies established so far, highlighting the need for further research to unify approaches [4,5,6]. In our previous study, a fatty kidney was defined as one with fat accumulation of more than 4% in the kidney, as detected by MRI [4].

Fatty kidney frequently coexists with metabolic disorders such as obesity, type 2 diabetes, hypertension, fatty liver disease, and metabolic syndrome. Obesity is a key risk factor that is strongly associated with ectopic lipid accumulation in the kidneys. Insulin resistance, oxidative stress, and chronic inflammation further exacerbate this condition, linking it to adverse renal and cardiovascular outcomes. Studies have demonstrated that individuals with fatty liver are significantly more likely to have fatty kidneys, suggesting a shared pathogenic mechanism involving ectopic fat deposition [7,8].

Although our understanding of fatty kidney has advanced, significant gaps remain regarding its epidemiology, pathophysiology, and implications across metabolic conditions [3,9]. This review aims to consolidate existing knowledge, applying imaging methodologies to investigate the prevalence of fatty kidney in metabolic disorders. By addressing these gaps, this study seeks to underscore the growing importance of fatty kidney as a clinical and research focus [3,9].

## 2. Methods

This systematic review study was registered at PROSPERO (CRD42024576368 on 4 August 2024) and was carried out following the Preferred Reporting Items for Systematic Review and Meta-Analyses (PRISMA) guidelines.

### 2.1. Search Strategy

A comprehensive and systematic search was conducted across four prominent databases, PubMed, Scopus, Web of Science, and Cochrane Library, up until August 2024. Our search query was (“fatty kidney” OR “renal steatosis” OR “renal fat” OR “kidney fat”) AND (“CT” OR “MRI” OR “computed tomography” OR “magnetic resonance imaging”).

### 2.2. Inclusion and Exclusion Criteria

The inclusion criteria were studies that 1. Evaluated fat content in the kidney using CT or MRI; 2. Were published in English; 3. Were original research articles or case series. All reviews, editorials, case reports, abstracts, and studies that were conducted in a language other than English were excluded.

### 2.3. Data Extraction

Data from the included studies were collected in a standardized data extraction sheet. These data included the first author’s name, year of publication, journal name, study design, sample size, sample demographic, sample comorbidities, imaging modality, origin of fat measured, method of fat evaluation, and fat percentage/volume measured. These data were reviewed by three authors (H.R, E.K, and S.A). In cases of discrepancies, a fourth author (E.G) was consulted to reach a consensus.

### 2.4. Risk of Bias

The Quality Assessment of Diagnostic Accuracy Studies (QUADAS-2) criteria were used to assess the risk of bias and applicability. The risk of bias was evaluated by the same three authors, and any discrepancies were resolved through discussion with a fourth author (E.G) to reach a consensus.

### 2.5. Data Analysis

A narrative synthesis was conducted to summarize the findings of the studies. Due to anticipated variability in fat measurement methods, participant comorbidities, and study designs, a meta-analysis was not planned. The results are presented in a tabular format, accompanied by a detailed discussion of the key insights.

## 3. Results

### 3.1. Search Results and Study Selection

**Figure 1:**
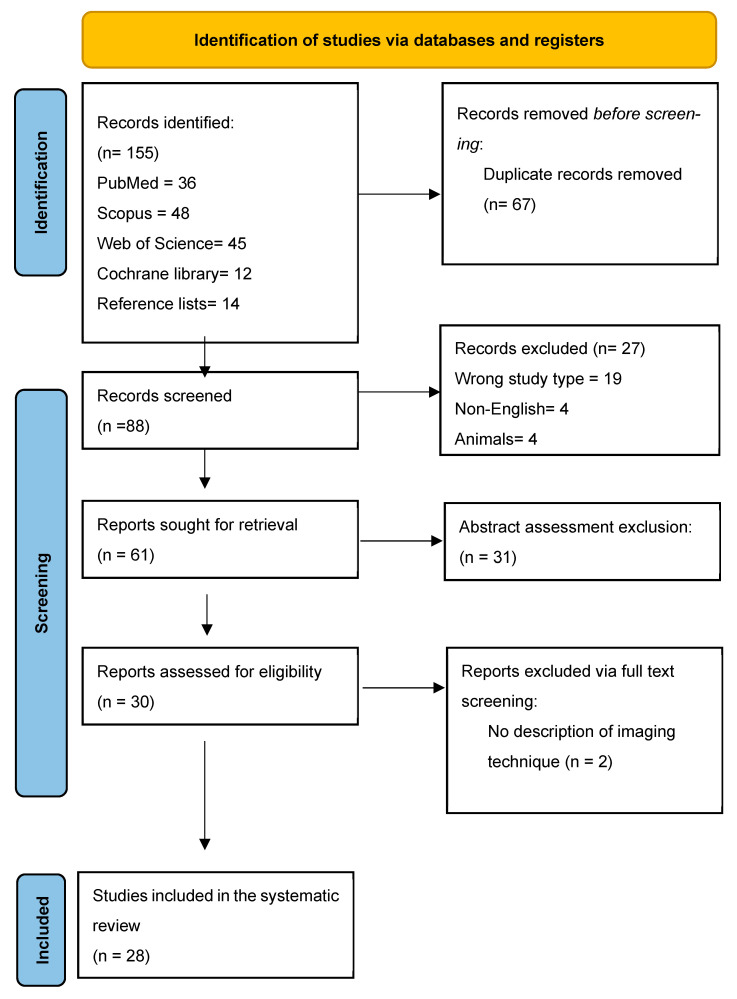
Identification of studies via databases and registers.

The initial search across the four databases yielded 141 studies (Figure 1). These were distributed as follows: PubMed (36 studies), Scopus (48 studies), Web of Science (45 studies), and Cochrane Library (12 studies). In addition, the reference lists of eligible articles were reviewed for any additional relevant studies. After removing duplicates (67), 88 studies remained for further inspection. Out of those, 27 were excluded: 19 unsuitable types, 4 non-English studies, and 4 animal studies. After the abstract assessment, 30 studies remained for full-text evaluation. These studies were further evaluated by three independent reviewers (H.R, E.K, and S.A) who excluded two studies for lacking descriptions of imaging techniques. The remaining 28 studies were included in the systematic review [4,10,11,12,13,14,15,16,17,18,19,20,21,22,23,24,25,26,27,28,29,30,31,32,33,34,35,36].

### 3.2. Risk of Bias and Quality Assessment

The QUADAS-2 tool was utilized to assess the risk of bias across multiple domains: patient selection, index test, reference standard, and flow and timing. Studies in which radiologists were not blinded to clinical data were classified as having a high risk of bias. The QUADAS-2 tool identified a high risk of bias and concerns regarding applicability in eight studies (28.57%) (Supplementary Table S1).

### 3.3. Overview of Included Studies

The studies included in the analysis were published between 2010 and 2024 (Table 1). Half of them were retrospective, seven were prospective, five were cross-sectional, and two were randomized controlled trials. Comparing imaging modalities, 75% of the studies used MRI to assess fat in the kidney, while only 25% used CT scans. Since CT scans cannot clearly identify fat in the kidney parenchyma, studies that used CT scans focused on the fat content in the renal sinus. Out of the twenty-one studies that used MRI, six measured the fat content only in the renal sinus, eleven measured only in the renal parenchyma, and four measured in both the sinus and the parenchyma. Details of these studies are summarized in Table 1. These studies included 6994 individuals aged between 27 and 61 years, with the proportion of females ranging from 11% to 65.3%. The renal fat fraction (FF) ranged from 0.4% to 55.3% and was higher among sub-populations with concomitant conditions such as diabetes mellitus, obesity, CKD, and hypertension (Table 1 and Table 2). Only three studies used the term “fatty kidney,” and each employed a different definition. Foster MC. et al. defined a fatty kidney as being in the top decile of fat accumulation by sex, corresponding to an area above 0.455 cm^2^ in women and above 0.71 cm^2^ in men [19]. Krievina G. et al. assessed the ratio of fat area in the renal sinus to the area of the left kidney, defining a fatty kidney in patients in the top quartile by sex [22]. Raphael H. et al. defined a fatty kidney as having more than 4% fat accumulation on MRI [4].

## 4. Association of Fatty Kidney with Specific Comorbidities

### 4.1. Diabetes Mellitus

The review includes eight studies of patients with type 2 diabetes mellitus in which renal fat content was assessed using MRI. Six of these studies did not include a control group; of those, three included only patients with diabetes [21,24,28]. Two studies measured fat in the renal sinus [24,26], while the remaining studies assessed fat in the renal parenchyma. Two studies compared patients with both diabetes and CKD [15,28]. Aydin et al. reported higher renal FFs in patients with both diabetes and CKD compared with controls [15]. Yang et al. [33] and Wang et al. [32] compared diabetic patients with and without nephropathy to a control group. Both studies found higher FFs in diabetic patients compared with controls and further increases in FFs among those with nephropathy compared with those without.

None of the studies provided a definition for “fatty kidney” or reported the proportion of patients classified as having fat in the kidney (Table 3).

### 4.2. Obesity

Eight studies that evaluated the association between fatty kidney and obesity were included in this review (Table 4). Half of the studies did not include a control group. Three studies assessed the fat contents in both the renal parenchyma and the renal sinus [30,31,35]. Sijens et al. conducted a study with a relatively small cohort of 36 patients, measured fat content using Dixon’s technique, and corrected it against MRS (magnetic resonance spectroscopy) [29].

Spurny et al. followed obese participants over a 50 week dietary intervention trial, investigating the effect of weight loss on renal fat content [30]. They measured fat in both the renal sinus and parenchyma, demonstrating a reduction in sinus fat but not in cortical fat following weight loss. These findings are consistent with those of Zelicha et al., who reported a significant linear decrease in renal sinus fat and a modest reduction in cortical fat across weight loss quartiles [35]. Both Spurny et al. and Zelicha et al. observed a correlation between reductions in renal sinus fat and improvements in fatty liver disease-Spurny et al. showed a decrease in liver fat, while Zelicha et al. reported improved hepatic biomarkers [30,35]. Lee et al. compared adipose tissue volumes in the periaortic, renal sinus, visceral, and subcutaneous compartments across four groups: non-obese without metabolic syndrome (n = 64), non-obese with metabolic syndrome (n = 25), obese without metabolic syndrome (n = 21), and obese with metabolic syndrome (n = 129). They found significantly higher fat volumes in all compartments among obese individuals compared with non-obese and among individuals with metabolic syndrome compared with those without, regardless of obesity status [23]. Gjela et al. evaluated four MRI methods for measuring the renal proton density fat fraction (PDFF) in obese and control participants [20]. All four methods demonstrated higher renal PDFF values in obese individuals compared with lean controls. Finally, Raphael et al. reported that obese patients had higher rates of both fatty liver and fatty kidney disease [4]. Obesity was identified as the sole independent risk factor for the co-occurrence of fatty liver and fatty kidney, with an adjusted odds ratio of 6.3.

### 4.3. Chronic Kidney Disease

Four studies examined the correlation between renal FF and CKD using Dixon’s MRI technique (Table 5). Aydin et al. assessed renal steatosis in both the cortex and medulla in CKD patients with and without diabetes [15]. They found that fat accumulation in the kidney increased proportionally with the CKD stage and reported higher cortical FFs in patients with both CKD and diabetes compared with those with CKD alone. Wang et al. and Yang et al. investigated the association between diabetic nephropathy and renal FF [32,33]. Among diabetic patients, Yang et al. [33] observed significantly higher FF values in those with nephropathy compared with those without. Similarly, Wang et al. [32] reported increased renal FFs in patients with microalbuminuria compared with normoalbuminuric patients and healthy controls (Table 5).

### 4.4. Hypertension

Two studies included in the review reported similar observations regarding the relationship between fatty kidneys and hypertension [17,25]. Moritz et al. found that patients with hypertension had a larger renal sinus fat (RSF) deposit compared with normotensive individuals [25]. They followed obese patients after bariatric surgery and observed a significant reduction in RSF along with a high rate of hypertension remission. Notably, greater reductions in RSF were associated with higher rates of hypertension remission.

Chugh et al. reported significantly higher RSF volumes in patients with stage II hypertension. In addition, RSF volume was positively associated with the number of prescribed antihypertensive medications, renal size, and serum creatinine levels [17].

### 4.5. The Association Between Fatty Liver and Fatty Kidney

The review included 11 studies that investigated the relationship between fatty liver and fatty kidney [4,10,14,17,18,26,27,29,30,34,36]. The definition of fatty liver varied across the studies. Three studies explicitly defined fatty liver using different modalities: CT [18], MRI [4], and histology [14]. The remaining studies referred to liver fat accumulation or hepatic fat fraction but did not specify a formal definition of fatty liver. Three studies found no correlation between the two conditions [14,27,29], and two studies reported associations that did not reach statistical significance [10,26]. Couch et al. measured liver fat but did not report any correlation with fatty kidney [17]. Five studies demonstrated a significant correlation between fatty liver and renal fat accumulation. Spurny et al. found a positive correlation between liver fat and both renal sinus fat and total kidney fat but no correlation with cortical kidney fat [30]. No correlation was found between kidney cortex fat and liver fat. Raphael et al. reported co-occurrence of fatty liver and fatty kidney in 6% of individuals and demonstrated a strong association between the two, with an odds ratio (OR) of 2.9 [4]. Similar findings were reported by Yıldız et al., who found a strong positive correlation between liver and kidney fat accumulation scores (Pearson correlation coefficient = 0.71, *p* < 0.001) [34]. Dogan et al., using CT, identified higher renal sinus fat diameters in patients with fatty liver, suggesting that RSF may serve as a useful adjunct marker for detecting hepatic steatosis in young adults [18]. Zhang et al. reported similar results using MRI, showing an association between RSF volume and fatty liver, and further demonstrated that renal parenchymal fat fraction was also correlated with liver fat accumulation [36].

## 5. Discussion

This systematic review highlights the increasing recognition of fatty kidney as an emerging clinical entity with strong associations with diabetes, obesity, CKD, hypertension, and fatty liver disease. Findings from multiple studies suggest that fat accumulation in the kidney is not merely an epiphenomenon of systemic metabolic dysfunction but rather a potential contributor to renal and cardiovascular pathology. Given the rising prevalence of metabolic disorders worldwide, the significance of fatty kidney as a possible early marker and risk factor for kidney disease progression warrants further investigation [19].

The studies included in this review demonstrate a wide range of reported prevalence of renal fat accumulation, which is likely due to differences in the methodologies used to assess this entity. Only studies that used CT or MRI to evaluate renal fat were selected, yet even within these imaging modalities, there is a lack of standardized criteria for diagnosing renal steatosis. Some studies quantified fat in the renal sinus, while others focused on the renal parenchyma. When reporting fat accumulation in the kidney, it is important to consider that measurement variability may arise because of differences in the anatomical sites being assessed. Unlike the kidney, the liver has a more uniform gross anatomy, which allows for more consistent and reproducible measurements. Notably, most studies did not define “fatty kidney” as a distinct clinical entity but rather described fat fraction percentages in various anatomical regions of the kidney. In our previous work, we proposed defining fatty kidney as a fat fraction equal to or greater than 4%.

Renal fat accumulation induces inflammation and fibrosis and ultimately may contribute to CKD. Several experimental studies have suggested that anti-inflammatory treatments may mitigate kidney damage related to fat accumulation. Renal lipid accumulation activates oxidative stress pathways, pro-inflammatory signaling, and the renin–angiotensin–aldosterone system. Renal lipotoxicity affects all major cell types in the kidney, including mesangial cells, podocytes, glomerular endothelial cells, and proximal tubular epithelial cells. Lipid accumulation, partly mediated via CD36, triggers the expression of several growth factors (e.g., TGF-β1, PDGF, and CTGF) and pro-inflammatory cytokines and chemokines (e.g., IL-1β, IL-6, MCP-1, NF-κβ, and TNF-α). These mediators contribute to leukocyte recruitment, mesangial expansion, glomerular endothelial dysfunction, podocyte loss, and tubular damage with disruption of the basement membrane [1].

Obesity is an important contributor to renal lipid accumulation, with multiple studies demonstrating an association between body mass index (BMI) and renal FF. Several studies have shown that renal PDFF measured using Dixon MRI is significantly higher in obese individuals compared with non-obese individuals, with BMI showing a strong independent correlation with renal FF [25]. MRI-based quantification of renal fat has consistently demonstrated increased renal lipid content in obese subjects, supporting the relationship between ectopic renal fat accumulation and metabolic dysfunction [15]. Additionally, research has confirmed that renal sinus fat fraction correlates significantly with BMI and that the degree of fat accumulation in the kidney increased progressively across higher BMI categories, suggesting that systemic adiposity plays a role in renal lipid deposition [4,22,35].

Across all included studies, overweight and obese individuals showed consistently higher levels of renal fat. This was particularly pronounced in individuals with metabolic syndrome.

Weight loss led to reductions in renal sinus fat. While significant decreases in renal sinus fat are observed following bariatric surgery and dietary interventions, changes in renal parenchymal fat are minimal [25]. This suggests that while visceral adipose deposits may respond to caloric restriction, intra-renal lipid accumulation in the parenchyma may persist despite systemic weight loss, potentially contributing to long-term renal dysfunction [35].

Several studies also reported an association between hepatic steatosis and renal fat accumulation, suggesting that ectopic fat deposition is a hallmark of metabolic syndrome and contributes to systemic inflammation, fibrosis, and irreversible organ damage.

In an animal model, it has been demonstrated that a fructose-rich diet induces features of metabolic syndrome and renal steatosis, resembling mechanisms observed in hepatic steatosis [37].

This review highlights the association between fatty kidney and diabetes, particularly in patients with diabetic nephropathy. Research indicates that patients with type 2 diabetes tend to have higher renal FF values than non-diabetics, with even greater lipid accumulation observed in those with nephropathy [25]. The underlying mechanisms may involve insulin resistance, oxidative stress, and lipid-induced mitochondrial dysfunction, which are known contributors to renal injury [22]. Additionally, chronic hyperglycemia has been linked to increased renal fat deposition, likely through the effects of advanced glycation end products (AGEs) [25]. Fatty kidney may be an early marker of kidney disease in diabetes, with microalbuminuria indicating early renal dysfunction. Its association with lipid accumulation and glomerular damage highlights the need for early detection and intervention [35].

Diabetes is a major driver of CKD, and lipotoxicity-induced inflammation may accelerate renal injury in these patients.

Fat deposition in the kidney has also been associated with hypertension. Studies have shown that hypertensive individuals tend to have more renal fat and weight loss is associated with improved blood pressure control and reduced renal fat.

In patients with CKD or diabetic nephropathy, the prevalence of renal fat accumulation was high. However, it remains unclear whether renal steatosis is a cause or consequence of kidney dysfunction. Regardless, it is evident that lipid accumulation promotes inflammation and exacerbates kidney damage. Research suggests that a higher renal FF is associated with declining eGFR and increased markers of renal injury, such as kidney injury molecule-1 (KIM-1) and fibroblast growth factor-21 (FGF-21) [22]. Moreover, renal sinus fat accumulation has been associated with increased renal vascular resistance and elevated blood pressure, indicating a potential mechanical and inflammatory role in CKD development [36]. In a study examining a large population cohort, higher renal sinus fat was independently associated with hypertension and CKD severity, reinforcing the concept that fat accumulation within the kidney contributes to progressive renal dysfunction [25].

Research indicates that patients with nephrolithiasis tend to have higher renal sinus fat volumes, suggesting that ectopic fat accumulation may contribute to kidney stone formation through altered renal hemodynamics and calcium metabolism [22]. This needs further research into whether fatty kidney predisposes individuals to kidney stone disease.

Interestingly, some studies reported a left-sided predominance of fat accumulation in the kidneys [16,27]. In our previous study, we also observed a greater tendency for fat to accumulate in the left kidney [4], a finding echoed by other researchers [16,27]. This may possibly be due to the anatomical phenomenon linked to differences in renal venous drainage. As the left renal vein is longer, traveling anterior to the aorta to drain into the inferior vena cava. While the reason remains unclear, this observation highlights the need to standardize measurements and potentially focus on the left kidney when assessing renal fat deposition.

**Figure 2:**
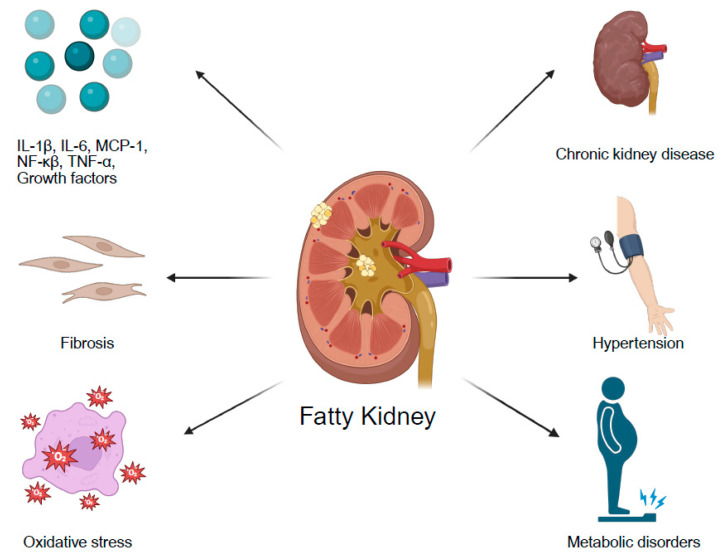
A schematic summary of the pathophysiology of fatty kidney and its associations with comorbidities. Created in https://BioRender.com (accessed on 10 June 2025).

Renal steatosis may serve as an early marker of kidney injury and is associated with systemic inflammation and increased cardiovascular risk (Figure 2). Early identification of patients with fatty kidney is crucial to enable close monitoring and early interventions, including weight loss and management of cardiovascular risk factors.

This systematic review has several limitations. First, the quality of the studies included in the review varies considerably. Using the QUADAS-2 tool, we identified a high risk of bias in several studies, which may limit the strength and broader applicability of their findings. Second, there is a lack of consistency in measuring renal fat and defining what constitutes a fatty kidney. In a previous study, we defined the accumulation of more than 4% fat in the kidney as indicative of a fatty kidney. This definition was arbitrary and set a relatively high fat threshold to confidently designate a kidney as fatty. To validate our definition, it is necessary to align the imaging findings with the histopathological results of the kidney. Prospective studies are needed to investigate the association between the percentage of fat deposition in the kidney and the development of kidney failure and cardiovascular morbidity.

To improve clinical practice and research, there is a need to establish clear diagnostic criteria and standardized measurement protocols for renal fat accumulation. Just as liver steatosis is routinely reported in abdominal CT and MRI evaluations, we propose that radiologists begin to report renal fat content in abdominal imaging studies. This will enhance early detection of subclinical renal disease and improve our understanding of the natural history of fatty kidney disease.

## Figures and Tables

**Table 1:** Studies included in the review.

Author, Year of Publication	Modality	Study Type	Patients, n	Age (Years)	Female Sex (%)	BMI, kg/m^2^, Median	Comorbidities
Hüseyin Aydın, 2023 [15]	MRI	Retrospective	88	Control 55.4 CKD 59.3	46.7%	N/A	CKD, Diabetes
Huali Tang, 2024 [31]	MRI	Prospective	103	Control 27 Obese 50.5	Control 41% Obese 77%,	Control 21.6 Obese 30.1	Obesity
Manuela Spurny, 2022 [30]	MRI	RCT	137	N/A	N/A	~31	Obesity, Fatty liver
Takeshi Yokoo, 2016 [10]	MRI	Retrospective	69	58	34.8%	30.6	Diabetes, Fatty liver
Mimoza Gjela, 2022 [20]	MRI	Retrospective Observational Case-control	42	Lean 44.4 Obese 46.5	Lean 57.1% Obese 67.8%	Lean 24 Obese 35.5	Obesity
Yan Shen, 2022 [28]	MRI	Cross-sectional Observational	189	57	34%	25.3	CKD, Diabetes
Meredith C. Foster, 2011 [19]	CT	Cross-sectional	2923	54	51	Non-fatty kidney 26.6 Fatty kidney 30.3	N/A
Yulin Hua, 2024 [21]	MRI	Retrospective	118	54	29.7%	24.9	Diabetes
Paul E Sijens, 2010 [29]	MRI, MRS	Prospective Observational	36	39	33.3%	27.5	Obesity, Fatty liver
Yuan-Cheng Wang, 2018 [32]	MRI	Prospective Observational	95	Control 61 Diabetic normoalbuminuric 57 Diabetic microalbuminuric 59	43.2%	Control 22.6 Diabetic normoalbuminuric 25.3 Diabetic microalbuminuric 25.2	Diabetes, CKD
Hadar Raphael, 2024 [4]	MRI	Retrospective	399	51	52.4%	24.6	Obesity, Fatty liver
Abdullah B. Yıldız, 2024 [34]	MRI	Retrospective Cross-sectional	51	34	51	26.4	Fatty liver
Chun Yang, 2023 [33]	MRI	Prospective	60	N/A	45	N/A	Diabetes, CKD
Ahmad A. Alhulail, 2022 [11]	MRS	Prospective	5	31	20%	25	N/A
Emrah Doğan, 2022 [18]	CT	Retrospective	92	30.2	20.7%	N/A	Fatty liver
Emilia Moritz, 2022 [25]	MRI	Prospective	120	Control 46 Obese 45	Control 78.3% Obese 91.9%	Control 23.4 Obese 41.5	Obesity, Hypertension
Gita Krievina, 2016 [22]	CT	Cross-sectional Observational	280	38.3	50%	27.9	N/A
Haroon L Chughtai, 2010 [16]	MRI	Cross-sectional Observational	205	69	49%	30	Hypertension
Ling Lin, 2023 [24]	MRI	Retrospective	93	56.9	50.5%	30.8	Diabetes
Ivan Ordulj, 2024 [27]	CT	Retrospective	302	49.5	28.2%	N/A	Fatty liver
Peng Lin, 2020 [12]	CT	Retrospective	232	47	50%	Control 20.8 Nephrolithiasis 22.4	Nephrolithiasis
Catharine A. Couch, 2022 [17]	MRI	Prospective Cross-sectional	116	29.2	52.6%	27.4	Fatty liver
Eun Ji Lee, 2021 [23]	CT	Retrospective	239	41.2	65.3%	By groups	Obesity
Mike Notohamiprodjo, 2020 [26]	MRI	Cross-sectional Observational	366	56.2	43.2	28.1	Diabetes, Fatty liver
Hila Zelicha, 2018 [35]	MRI	Observational	278	47.8	11%	31	Obesity
Yoko Murakami, 2015 [13]	CT	Retrospective	189	66.7	28%	CAC ≤ 10 25.1 CAC > 10 24	Coronary artery disease
Ilkay S. Idilman, 2015 [14]	MRI	Retrospective	41	47	51.2%	29.9	Fatty liver
Qin-He Zhang, 2023 [36]	MRI	Retrospective	126	56	63.5%	23.6	Fatty liver

BMI = body mass index, N/A = not available, CKD = chronic kidney disease, MRI = magnetic resonance imaging, CT = computed tomography, MRS = magnetic resonance spectroscopy, CAC = coronary artery calcium score.

**Table 2:** Main findings of the included studies.

Author, Year of Publication	Study Population	Measured Area	Result- Renal Fat			Patients with Fatty Kidney, (%)
			Control	Disease	All	
Hüseyin Aydın, 2023 [15]	CKD, Diabetes	Parenchyma	Cortex 5.7% FF Medulla 4.5% FF	CKD with diabetes: cortex 8.1% FF, medulla 6.9% FF CKD without diabetes: cortex 6.6% FF, medulla 5.8% FF CKD: cortex 7.2% FF, medulla 6.3% FF	N/A	N/A
Huali Tang, 2024 [31]	Obesity	Parenchyma, Sinus	1.38% FF, 8.42 cm^3^	2.01% FF, 17.41 cm^3^	N/A	N/A
Manuela Spurny, 2022 [30]	Obesity, Weight loss quartiles	Parenchyma, Sinus	N/A	Cortex: Q1 3.6% FF, Q2 3.2% FF, Q3 2.9% FF, Q4 3.4% FF [avg 3.3% FF] Sinus: 55.3% FF, 49.2% FF, 49.1% FF, 49.4% FF [avg 50.8% FF]	N/A	N/A
Takeshi Yokoo, 2016 [10]	Diabetes	Parenchyma	0.79% FF	2.18% FF	1.33% FF	N/A
Mimoza Gjela, 2022 [20]	Obesity	Parenchyma	method 1 1.8% FF, method 2 2% FF, method 3 0.4% FF, method 4 1.4% FF	2.3% FF, 2.4% FF, 0.5% FF, 1.6% FF	N/A	N/A
Yan Shen, 2022 [28]	Diabetes with or without CKD	Parenchyma	Right kidney: 1.87% FF Left kidney: 2.23% FF	Right kidney: 2.17% FF Left kidney: 2.49% FF	N/A	N/A
Meredith C. Foster, 2011 [19]	N/A	Sinus	0.31 cm^2^	N/A	N/A	30.1%
Yulin Hua, 2024 [21]	Diabetes	Parenchyma	N/A	4.89% FF	N/A	N/A
Paul E Sijens, 2010 [29]	Obesity	Parenchyma	0.64% FF	1.35% FF	0.7% FF	N/A
Yuan-Cheng Wang, 2018 [32]	Diabetes with normoalbuminuria or microalbuminuria	Parenchyma	4.3% FF	Normoalbuminuria 4.7% FF Microalbuminuria 5.6% FF	N/A	N/A
Hadar Raphael, 2024 [4]	Obesity	Parenchyma	N/A	N/A	N/A	18.6%
Abdullah B. Yıldız, 2024 [34]		Parenchyma	N/A	N/A	N/A	N/A
Chun Yang, 2023 [33]	Diabetes with or without Diabetic Nephropathy	Parenchyma	1.11% FF	Diabetes 1.52% FF Diabetic nephropathy 1.99% FF	N/A	N/A
Ahmad A. Alhulail, 2022 [11]		Parenchyma	1.48% FF	N/A	N/A	N/A
Emrah Doğan, 2022 [18]	Fatty liver	Sinus	9.3 mm	12.5 mm	N/A	N/A
Emilia Moritz, 2022 [25]	Obesity	Sinus	1.8 cm^2^	2.3 cm^2^ (avg two kidneys)	N/A	N/A
Gita Krievina, 2016 [22]		Sinus	Right kidney 1.07 cm^3^ Left kidney 2.5 cm^3^	N/A	N/A	28.9%
Haroon L Chughtai, 2010 [16]		Sinus	4.2 cm^3^	N/A	N/A	28.9%
Ling Lin, 2023 [24]	Diabetes	Sinus	N/A	N/A	Left kidney West European 18.2 cm^3^ Left kidney South Asian 12.3 cm^3^	N/A
Ivan Ordulj, 2024 [27]		Sinus	N/A	N/A	Right kidney: 2.56 cm^2^ Left kidney: 2.83 cm^2^	N/A
Peng Lin, 2020 [12]	Nephrolithiasis	Sinus	Right kidney 3.34 cm^3^ Left kidney 4.56 cm^3^	Right kidney 4.14 cm^3^ Left kidney 5.47 cm^3^	N/A	N/A
Catharine A. Couch, 2022 [17]		Sinus	1.05 cm^3^	N/A	N/A	N/A
Eun Ji Lee, 2021 [23]	Obesity, Metabolic syndrome	Sinus	N/A	N/A	N/A	N/A
Mike Notohamiprodjo, 2020 [26]	Prediabetes, Diabetes	Sinus	22.2 mL	Prediabetes 32 mL Diabetes 34.5 mL	26.2 mL	N/A
Hila Zelicha, 2018 [35]	Obesity	Parenchyma, Sinus	N/A	N/A	Parenchyma 7.9% FF Sinus 2.7 cm^2^	N/A
Yoko Murakami, 2015 [13]	Coronary artery disease with CAC score > 10	Sinus	5.60 cm^3^	7.48 cm^3^	7.05 cm^3^	N/A
Ilkay S. Idilman, 2015 [14]	NAFLD	Parenchyma, Sinus	N/A	N/A	Cortex 1.7% FF Sinus 51% FF	N/A
Qin-He Zhang, 2023 [36]	Men vs. Women	Sinus	Men: Right kidney 28.3% FF, 26.8 cm^3^ Left kidney 31.2% FF, 31.6 cm^3^	Women: Right kidney 23.8% FF, 21.4 cm^3^ Left kidney 27.9% FF, 26 cm^3^	Right kidney 25.4% FF, 23.4 cm^3^ Left kidney 29% FF, 28 cm^3^	N/A

CKD = chronic kidney disease, N/A = not available, FF = fat fraction, NAFLD = non-alcoholic fatty liver disease.

**Table 3:** Studies that evaluated renal fat in diabetic patients.

First Author	Comorbidities	Measured Area	Result- Renal Fat			
			Control	Diabetes	With Comorbidity	All
Hüseyin Aydın [15]	CKD	Cortex	5.7% FF		8.1% FF	N/A
		Medulla	4.5% FF		6.9% FF	N/A
Takeshi Yokoo [10]	N/A	Parenchyma	0.79% FF	2.18% FF	N/A	1.33% FF
Yan Shen [28]	CKD	Right parenchyma	N/A	1.87% FF	2.17% FF	N/A
		Left Parenchyma	N/A	2.23% FF	2.49% FF	N/A
Yulin Hua [21]	N/A	Parenchyma	N/A	4.89% FF	N/A	N/A
Yuan-Cheng Wang [32]	Normoalbuminuria	Parenchyma	N/A	4.3% FF	4.7% FF	N/A
	Microalbuminuria	Parenchyma	N/A	4.3% FF	5.6% FF	N/A
Chun Yang [33]	Diabetic Nephropathy	Parenchyma	1.11% FF	1.52% FF	1.99% FF	N/A
Ling Lin [24]	N/A, (West European)	Left sinus	N/A	18.2 cm^3^	N/A	N/A
	N/A (South Asian)	Left sinus	N/A	12.3 cm^3^	N/A	N/A
Mike Notohamiprodjo [26]	Prediabetes	Sinus	22.2 mL	34.5 mL	32 mL	26.2 mL

CKD = chronic kidney disease, N/A = not available, FF = fat fraction.

**Table 4:** Studies in patients with obesity.

First Author	Comorbidities	Measured Area	Result- Renal Fat			
			Control	Obese	With Comorbidity	All
Huali Tang [31]	N/A	Parenchyma	1.38% FF	2.01% FF	N/A	N/A
		Sinus	8.42 cm^3^	17.41 cm^3^	N/A	N/A
Manuela Spurny [30]	Weight loss quartiles	Cortex	N/A	Q1 3.6% FF, Q2 3.2% FF, Q3 2.9% FF, Q4 3.4% FF, Avg 3.3% FF	N/A	N/A
		Sinus	N/A	55.3% FF, 49.2% FF, 49.1% FF, 49.4% FF, Avg 50.8% FF	N/A	
Mimoza Gjela [20]	N/A	Parenchyma	method 1 1.8% FF, method 2 2% FF, method 3 0.4% FF, method 4 1.4% FF	2.3% FF, 2.4% FF, 0.5% FF, 1.6% FF	N/A	N/A
Paul E Sijens [29]	N/A	Parenchyma	0.64% FF	1.35% FF	N/A	0.7% FF
Hadar Raphael [4]	N/A	Parenchyma	N/A	N/A	N/A	N/A
Emilia Moritz [25]	N/A	Average of two kidneys Sinus	1.8 cm^2^	2.3 cm^2^	N/A	N/A
Eun Ji Lee [23]	N/A	Sinus	N/A	N/A	N/A	N/A
Hila Zelicha [35]	N/A	Parenchyma	N/A	N/A	N/A	7.9% FF
		Sinus	N/A	N/A	N/A	2.7 cm^2^

N/A = not available, FF = fat fraction.

**Table 5:** Studies in patients with chronic kidney disease.

Author	Comorbidities	Measured Area	Result- Renal Fat			
			Control	CKD	With Comorbidity	All
Hüseyin Aydın [15]	Diabetes	Cortex	5.7% FF	7.2% FF without diabetes: 6.6% FF	8.1% FF	N/A
		Medulla	4.5% FF	6.3% FF without diabetes: 5.8% FF	6.9% FF	N/A
Yan Shen [28]	Diabetes	Right parenchyma	N/A	N/A	2.17% FF	N/A
		Left Parenchyma	N/A	N/A	2.49% FF	N/A
Yuan-Cheng Wang [32]	Diabetes	Parenchyma	N/A	N/A	5.6% FF	N/A
Chun Yang [33]	Diabetes	Parenchyma	1.11% FF	N/A	1.99% FF	N/A

CKD = chronic kidney disease, N/A = not available, FF = fat fraction.

## Supplementary Materials

The following supporting information can be downloaded at: https://www.mdpi.com/article/10.3390/jcm14124305/s1, Table S1: Risk of bias of included studies.
