# Supplementary material for: Renal Fat Accumulation Assessed by MRI or CT and Its Association with Clinical and Metabolic Disorders: A Systematic Imaging Review
Source: J Clin Med. 2025 Jun 17;14(12):4305. doi: 10.3390/jcm14124305 (PMC12194363; doi:10.3390/jcm14124305)
Supplement: Supplementary file 1 [file jcm-14-04305-s001.zip › jcm-3639098-supplementary.pdf]

Study	Risk of bias				Applicability concerns		
	Patient selection	Index test	Reference standard	Flow and timing	Patient selection	Index test	Reference standard
Hüseyin Aydın, 2023 [15]	🟢	🟡	🟢	🟢	🟢	🟢	🟢
Huali Tang, 2024 [31]	🟢	🟢	🟢	🟢	🟢	🟢	🟢
Manuela Spurny, 2022 [30]	🟢	🟢	🟡	🟢	🟢	🟢	🟢
Takeshi Yokoo, 2016 [10]	🟢	🟡	🟡	🟢	🟢	🟢	🟢
Mimoza Gjela, 2022 [20]	🟢	🔴	🔴	🔴	🟢	🟢	🔴
Yan Shen, 2022 [28]	🟢	🟡	🟡	🟢	🟢	🟢	🟢
Meredith C. Foster, 2011 [19]	🟢	🟡	🟡	🟢	🟢	🟢	🟢
Yulin Hua, 2024 [21]	🟢	🟡	🟡	🟢	🟢	🟢	🟢
Paul E Sijens, 2010 [29]	🟢	🟡	🔴	🟢	🟢	🟢	🔴
Yuan-Cheng Wang, 2018 [32]	🟢	🟡	🟡	🟢	🟢	🟢	🟢
Hadar Raphael, 2024 [4]	🟢	🟡	🟡	🟢	🟢	🟢	🟢
Abdullah B. Yıldız, 2024 [34]	🟢	🟡	🟡	🟢	🟢	🟢	🟢
Chun Yang, 2023 [13]	🟢	🟡	🟡	🟢	🟢	🟢	🟢
Ahmad A. Alhulail, 2022 [33]	🟢	🟢	🔴	🟢	🔴	🔴	🔴
Emrah Doğan, 2022 [18]	🟢	🟡	🟡	🟢	🟢	🟢	🟡
Emilia Moritz, 2022 [25]	🟢	🟡	🟡	🟢	🟢	🟢	🟢
Gita Krievina, 2016 [22]	🟢	🟡	🟡	🟢	🟢	🟢	🟢
Haroon L Chughtai, 2010 [16]	🟢	🟡	🟡	🟢	🟢	🟢	🟢
Ling Lin, 2023 [24]	🟢	🟡	🟡	🟢	🟢	🟢	🟢
Ivan Ordulj, 2024 [27]	🟢	🟢	🟢	🟢	🟢	🟢	🟢
Peng Lin, 2020 [12]	🟢	🟡	🟡	🟢	🔴	🟢	🔴
Catharine A. Couch, 2022 [17]	🟢	🟡	🟡	🟢	🟢	🟢	🔴

Eun Ji Lee, 2021 [23]	🟢	🟢	🟡	🟢	🟢	🟢	🟢
Mike Notohamiprodjo, 2020 [26]	🟢	🟢	🟡	🟢	🟢	🟢	🟢
Hila Zelicha, 2018 [35]	🟢	🟢	🟡	🟢	🟢	🟢	🟢
Yoko Murakami, 2015 [13]	🟢	🟡	🟡	🟢	🟢	🟢	🔴
Ilkay S. Idilman, 2015 [14]	🟢	🟡	🟡	🟢	🔴	🟢	🔴
Qin-He Zhang, 2023 [36]	🟢	🟡	🟡	🟢	🔴	🟢	🟢

Green- low, yellow- unclear, red- high
